# Aging, longevity, and healthy aging: the public health approach

**DOI:** 10.1007/s40520-025-03021-8

**Published:** 2025-04-17

**Authors:** Vincenza Gianfredi, Daniele Nucci, Flavia Pennisi, Stefania Maggi, Nicola Veronese, Pinar Soysal

**Affiliations:** 1 Department of Biomedical Sciences for Health, University of Milan, Milan, Italy; 2 Struttura Semplice Dipartimentale Igiene Alimenti e Nutrizione, Dipartimento di Igiene e Prevenzione Sanitaria, Agenzia di Tutela della Salute (ATS) Brescia, Viale Duca degli Abruzzi, 15, Brescia, 20124 Italy; 3 National PhD Programme in One Health Approaches to Infectious Diseases and Life Science Research, Department of Public Health, Experimental and Forensic Medicine, University of Pavia, Pavia, 27100 Italy; 4 Vita-Salute San Raffaele University, Milan, Italy; 5 Consiglio Nazionale delle Ricerche, Neuroscience Institute, Padova, Italy; 6 Saint Camillus International University of Health Sciences, Rome, Italy; 7 Department of Geriatric Medicine, Faculty of Medicine, Bezmialem Vakif University, Istanbul, Turkey

**Keywords:** Healthy aging, Longevity, Aging, Senescence

## Abstract

**Background:**

Population aging is one of the most significant global demographic changes of the 21st century, driven by increased life expectancy and declining fertility rates. This phenomenon presents both achievements and challenges for public health systems worldwide.

**Aims:**

On the one hand, advances in healthcare and socio-economic conditions have contributed to longer lives and improved quality of life for older adults. On the other hand, aging populations are increasingly affected by chronic diseases, greriatric syndromes, and multimorbidity, leading to greater healthcare demands and higher associated costs.

**Methods:**

This manuscript explores evidence on regards of the impact of aging on healthcare and economic systems, emphasizing the need for a paradigm shift toward healthy aging.

**Results:**

Healthy aging, as defined by the World Health Organization, focuses on the maintenance of intrinsic capacity, physical, mental, and social well-being throughout life. It highlights the importance of preventive healthcare, proper nutrition, and regular physical activity in delaying the onset of chronic conditions and maintaining functional independence. Furthermore, the manuscript addresses the challenges faced by healthcare infrastructures and pension systems as they adapt to aging populations, with particular attention to the strain caused by workforce shortages and the rising need for long-term care.

**Discussion:**

A coordinated public health approach is essential to promote healthy aging and mitigate the economic and societal impacts of population aging.

**Conclusions:**

This paper underscores the need for integrated health policies and multidisciplinary care models to ensure that longer life expectancy is accompanied by better quality of life for older individuals.

## Introduction

In scientific literature, there are numerous definitions of aging, each of which can be considered valid. However, many of these definitions seem limiting as they often focus solely on one aspect of the multifaceted nature of aging. For example, some definitions emphasize the biological decline associated with aging, such as the progressive loss of function or the persistent decline in age-specific fitness [1]. Other definitions, instead, focus on the age-related increase in mortality, considering the growing risk of death with advancing age [1]. Nevertheless, as already mentioned, focusing only on one of these aspects is limiting. On the contrary, the definition proposed by the World Health Organization (WHO) encompasses both biological and functional aspects [2]. It considers the accumulation of a wide variety of molecular and cellular damage over time (biological aspects), but simultaneously considers the gradual decline in physical and mental capacities, which ultimately increases the risk of illness and, consequently, death.

In this context, aging can be seen both as a great achievement and, at the same time, as a great challenge for systems. It is a significant achievement because it represents the result of increased life expectancy, thus higher longevity and a better quality of life. However, aging also represents a challenge, particularly due to the increase in morbidity and multimorbidity rates. This phenomenon requires greater allocation of resources in healthcare systems and calls for a reassessment of workforce distribution and pension systems, where a large percentage of retirees is supported by an ever-decreasing number of younger workers. Balancing these achievements and challenges is essential to effectively managing the impact of aging on both individuals and society. In this light, this paper aims to provide an overview of the issue of population aging, particularly by exploring its implications for public health, economic, and social systems.

### The global aging phenomenon

The aging of the global population represents one of the most significant demographic and social changes of the 21st century [3]. Rapid advances in medical science, public health (such as sanitation, vaccinations, antibiotics, and improvements in maternal and child health), and socioeconomic development have collectively contributed to an unprecedented increase in human longevity. For example, global life expectancy at birth increased from 46 years in 1950 to over 70 years in 2023 [4]. Despite these advances, life expectancy varies considerably across countries, with high-income countries such as Japan, Italy and Switzerland having average life expectancies exceeding 80 years, while many low-income nations, particularly in sub-Saharan Africa, have life expectancies below 60 years [5]. This means that, despite differences in life expectancy between countries, every country in the world is experiencing growth both in terms of the number and proportion of older people in their populations, contributing to the overall increase in the global population, with a larger proportion of older adults [5].

As a result, the number of individuals aged 60 and over is growing rapidly. By 2050, it is estimated that there will be more than 2.1 billion people aged 60 and over, representing over 21% of the global population [6]. The global aging trend is primarily driven by the general decline in fertility rates [7], improvements in healthcare systems [8, 9], and consequently, better diagnosis and treatment of diseases, as well as their prevention [10], and significant reductions in mortality from infectious diseases in favor of chronic-degenerative diseases—factors that have contributed to increasing life expectancy worldwide [11].

However, as societies age, the implications for public health, healthcare systems, and economies become increasingly complex. Longer life expectancy is frequently accompanied by an increase in chronic diseases such as cardiovascular diseases, cancer, diabetes, and neurodegenerative conditions such as Alzheimer’s disease [12]. These non-communicable diseases now represent the majority of deaths worldwide and disproportionately affect older populations [11]. Furthermore, as people live longer, many experience multimorbidity, that is, the coexistence of two or more chronic conditions, which complicates treatment and care management, leading to higher healthcare costs and increased demand for long-term care [11, 13, 14].

In many high-income countries, the rise in older populations with disabilitiy has led to increased healthcare needs, resulting in higher healthcare costs, alongside general staff shortages and tensions in pension systems [15, 16]. Around the world, 1.3 billion people, or 16% of the population, are living with significant disabilities, according to the WHO [17]. Meanwhile, middle- and low-income countries are experiencing similar demographic changes, though at a much faster pace, putting additional pressure on healthcare infrastructures that may already be underdeveloped or underfunded [18].

Indeed, while aging is an inevitable physiological process characterized by progressive physiological changes leading to a decline in biological functions and an increased risk of illness and mortality, the way individuals age varies significantly depending on factors such as genetics, lifestyle, and access to healthcare [19]. Social inequalities also play a significant role in how individuals experience aging. Factors such as lower socioeconomic status, limited access to healthcare, and educational disparities are linked to poorer health outcomes in older adults [10]. Therefore, while aging has historically been associated with the onset of chronic diseases and a decline in quality of life, contemporary research highlights that aging does not necessarily mean illness or disability [20, 21]. On the contrary, the focus has shifted toward promoting well-being and functional independence throughout life. This paradigm shift is aligns with the World Health Organization’s vision for the Decade of Healthy Aging (2021–2030) [22], which emphasizes a comprehensive approach to aging that goes beyond the absence of disease and focuses on the ability of older individuals to maintain physical, mental, and social well-being [23]. The new concept of “healthy aging” has emerged as a critical goal for public health systems worldwide, aiming to promote the inclusivity, dignity, and full participation of older adults within their communities, thereby supporting economic and systemic sustainability [24]. Healthy aging, as outlined by the WHO, refers to the process of developing and maintaining functional abilities that enable well-being in older age, advocating for supportive environments that empower individuals to live life to their fullest potential regardless of age [22]. The challenges posed by an aging population are multifaceted and require coordinated efforts across various sectors, including healthcare, social services, and public policies. A key challenge is ensuring that longer life expectancy is accompanied by better quality of life and functional independence for older people [15, 18]. The concept of healthy aging seeks to address this challenge by promoting a holistic approach focused on maintaining and enhancing individuals’ intrinsic capacity as they age.

## Population aging from a public health perspective

**Fig. 1:**
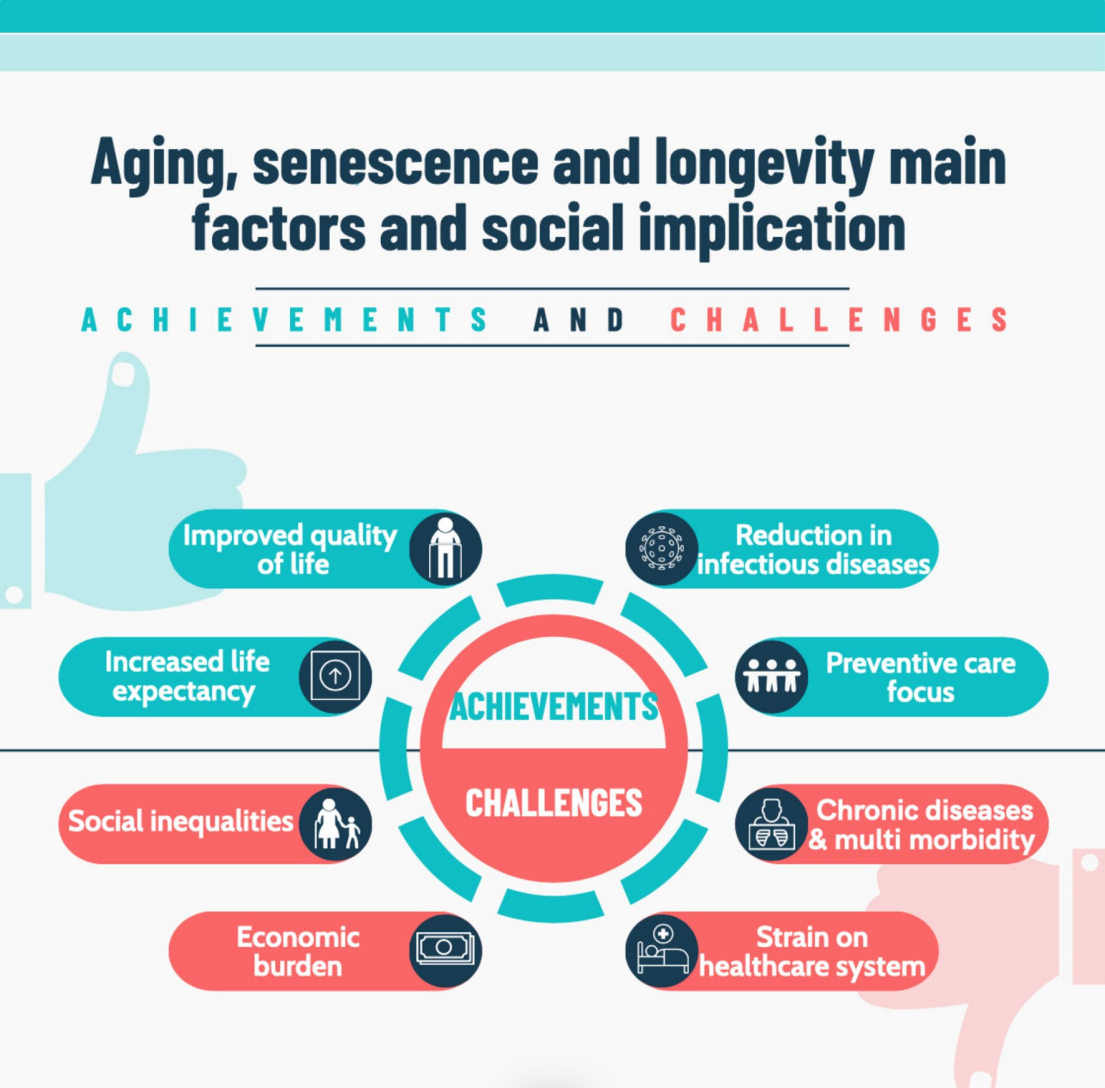
Achievements and challenges of aging: key factors and social implications

As previously mentioned, aging is seen from a public health perspective as both an achievement and a challenge (Fig. 1) [15, 18, 25]. Several key factors have contributed to the increase in life expectancy, and therefore to the longevity of populations. Among these, socio-political stability plays an important role. Countries with stable political environments tend to have higher life expectancies, as peace and economic growth create the conditions necessary to improve healthcare and social support systems [26]. At the same time, improvements in healthcare, thanks to advances in medical technology, expanded access to healthcare, and better management of infectious diseases first, and chronic diseases later, have contributed to increased life expectancy [27]. Ensuring access to clean water and improved sanitation conditions has played a crucial role in reducing the burden of infectious diseases, especially today, in low- and middle-income countries. Ensuring higher levels of education enables the adoption of healthier lifestyles with better health outcomes, as individuals with more education tend to engage in health-promoting behaviors and seek medical care when necessary [28]. Better nutrition and access to healthcare are closely linked to economic development, with wealthier countries generally exhibiting higher life expectancies. These factors collectively contribute to the demographic transition, wherein populations shift from high birth and death rates to low birth and death rates [29]. The phenomenon of demographic transition is accompanied by an epidemiological transition.

The epidemiological transition refers to the shift in the leading causes of death from infectious to non-communicable diseases (NCDs). Historically, populations were afflicted by high rates of infectious diseases such as smallpox, tuberculosis, and cholera, which were the primary causes of mortality. However, with the advent of vaccines, antibiotics, and improved sanitation conditions, the incidence of these diseases has drastically decreased, particularly in high-income countries [30]. Consequently, the leading causes of death have shifted to NCDs, which now account for 41 million deaths each year, representing 74% of all global deaths. Among these, 17 million people die prematurely—before the age of 70—with 86% of these early deaths occurring in low- and middle-income countries. Overall, 77% of all NCD-related deaths are concentrated in these regions. Cardiovascular diseases remain the most prevalent, causing 17.9 million deaths annually, followed by cancers (9.3 million), chronic respiratory diseases (4.1 million), and diabetes (2.0 million, including kidney disease caused by diabetes) [31].This transition is closely linked to population aging, as NCDs are more common in older individuals [11].

However, the aging population presents an additional challenge, represented not only by the higher prevalence of chronic diseases but also by multimorbidity [11]. Multimorbidity, defined as the coexistence of two or more chronic conditions, presents significant challenges for healthcare systems, as individuals with multiple chronic conditions often require more complex, resource-intensive care. As individuals age, they are more likely to develop multiple chronic conditions, which can complicate treatment and increase the burden on healthcare systems [32]. The public health challenge lies in balancing the need to support the growing older population while managing the associated healthcare costs and ensuring that resources are allocated effectively. This requires a shift in focus from treating diseases in isolation to promoting health and well-being throughout the life course, with an emphasis on prevention, early intervention, and maintaining functional abilities [33]. Managing multimorbidity in older adults requires an integrated approach to care. Multidisciplinary care teams, including geriatricians, dietitians, mental health professionals, and social workers, are essential for addressing the complexities of multiple chronic conditions [34]. Implementing chronic care models that emphasize coordination between primary care providers and specialists can reduce care fragmentation and improve outcomes.

The natural aging process, the onset of geriatric conditions and syndromes, and the presence of multimorbidity are all independently linked to functional decline. However, when these factors coexist, their impact is amplified, accelerating the deterioration of physical and cognitive function. Chronic diseases exacerbate this decline, progressively impairing an individual’s ability to perform activities of daily living (ADLs) independently and safely within their home and community [35, 36]. The deterioration of these abilities has been linked to higher rates of mortality, increased healthcare utilization and escalating healthcare costs [36]. Beyond its physical consequences, functional decline also exerts a profound psychosocial impact. Patients frequently struggle with the inability to return to their previous lifestyle or fulfill the life they had envisioned, often experiencing “chronic sorrow”—a persistent and recurrent grief response to the continuous personal and social losses associated with chronic illness or disability [37]. Studies indicate that increased dependency not only intensifies negative emotions but also diminishes self-efficacy, a critical psychological factor for adapting and coping effectively with chronic conditions [38]. As a result, individuals facing functional decline are at heightened risk for social isolation, reduced participation in community and social activities, and an overall decline in quality of life [39–41]. Addressing functional decline through early interventions, rehabilitation, and preventive strategies is therefore crucial in mitigating its adverse effects and preserving independence in aging populations.”.

## The impact of aging on healthcare and pension systems

With population aging, the intuitive assumption is that increasing rates of multimorbidity will lead to higher healthcare costs [42]. As mentioned earlier, an aging population requires the reallocation of resources within healthcare systems. However, when we compare life expectancy, birth rates, and government healthcare spending across different European (and non-EU) countries, a more nuanced picture emerges. For example, countries like Spain and Italy, where life expectancy is high (84.05 and 84.20 respectively) [43], do not necessarily have higher healthcare costs [18]. This suggests that other factors, such as national and per capita income, service prices, healthcare system model, and treatment intensity, play a more significant role in determining healthcare expenditures. This indicates that aging itself is not the direct cause of rising healthcare costs. Instead, the complexity associated with aging, particularly multimorbidity, often accompanies it, significantly contributing to these costs [44]. Economic analyses have shown that the increase in healthcare expenditures is not attributable to longevity per se [45, 46]. Rather, most healthcare spending is concentrated in the final years of life (end-of-life), during periods of severe illness, rather than during the additional years of relatively healthy life that result from increased life expectancy [44, 46]. This impact can be quantitatively assessed through health expectancy indicators, such as disability-free life expectancy (DFLE), which provides a measure of the proportion of life spent without significant disability or chronic illness. DFLE serves as a critical metric in evaluating the economic and social implications of population aging, offering a more precise understanding of the healthcare and long-term care needs associated with extended lifespans. According to the World Health Organization (WHO) Global Health Observatory, in most high-income countries, the gap between total life expectancy and DFLE is approximately 10 years, with an even greater discrepancy observed in low- and middle-income regions [47]. This finding underscores that while people are living longer, a substantial proportion of those additional years is characterized by declining health, increasing the demand for medical services, long-term care, and social support systems. From a public health and policy perspective, DFLE is a valuable indicator for guiding national strategies on aging, as it enables policymakers to anticipate healthcare and social care service needs more effectively [48]. The financial impact encompasses direct healthcare expenditures, including hospitalizations, pharmacological treatments, and assistive technologies, as well as long-term institutional and home-based care. Furthermore, a significant portion of this burden is borne by families and informal caregivers, highlighting the need for sustainable health and social care policies that promote healthy aging and reduce disability in later life.

Population aging also has significant implications for pension systems and workforce allocation. As the proportion of older adults in the population increases, the ratio of retirees to the working population becomes increasingly imbalanced [49]. This has raised concerns about the sustainability of pension systems, as fewer workers are available to support a growing number of retirees. To address this challenge, many countries are considering raising the retirement age, encouraging older adults to remain in the workforce longer, and promoting policies that support intergenerational equity. Moreover, workforce planning is necessary to ensure that healthcare systems have the capacity to meet the needs of the older population, including recruiting and retaining healthcare professionals [50].

Additionally, it is important to consider the effects of raising the retirement age on the health of older workers. Studies have shown that the impact of retirement on health is complex and influenced by factors such as pre-existing health conditions, financial stability, and the work environment [51]. Research has demonstrated that health issues significantly affect retirement decisions, particularly among older adults. Specifically, those experiencing physical or mental decline tend to retire early due to health problems [52]. In contrast, those who choose to work longer and delay retirement are generally in better health. This phenomenon is referred to as the “healthy worker effect” [53]. This phenomenon occurs when individuals who remain in the workforce are generally healthier than those who leave early due to illness or disability. A recent study comparing employed individuals and recently retired individuals aged 60 to 64, showed higher prevalence ratios for health problems such as functional limitations, heart disease, stroke, and chronic obstructive pulmonary disease among retirees, indicating that retirees generally had worse health compared to those who continued to work [54]. Furthermore, a recent systematic review with meta-analysis, which explored the association between retirement and the risk of depression, demonstrated that retirement reduces the risk of depression by approximately 20% compared to individuals over 60 who remain in the workforce [55]. However, the association between retirement and depression risk remains a topic of debate. While some studies suggest a potential protective effect of retirement on mental health [55], others indicate that the transition to retirement may not universally reduce the risk of depression and may even be associated with an increased risk under certain circumstances [56]. This apparent contradiction may be explained by the fact that the transition from employment to retirement can initially increase the risk of depression, a phenomenon that tends to diminish over the years of retirement. Therefore, these discrepancies highlight the need to consider individual, social, and occupational factors when evaluating the impact of retirement on mental health. In this perspective, postponing the retirement age, as foreseen in some pension reforms, could have negative effects on the health of workers who have experienced difficult working conditions before retirement.

## Public health approaches to healthy aging

**Fig. 2:**
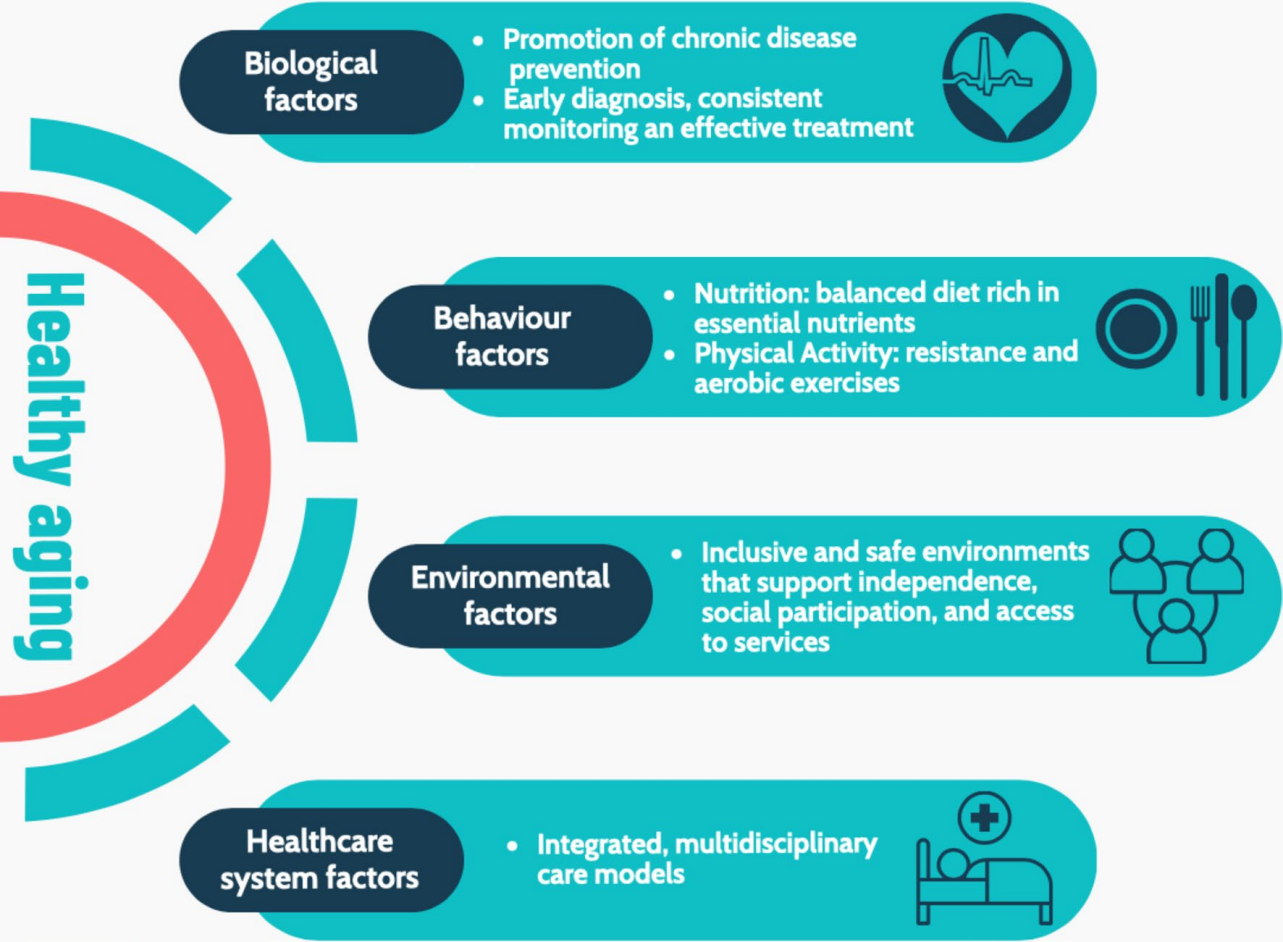
Determinants of healthy aging: biological, behavioral, environmental, and healthcare system factors

Public health approaches aimed at promoting healthy aging emphasize the importance of prevention, early intervention, and the promotion of healthy lifestyles throughout the life course (Fig. 2). The WHO “Decade of Healthy Aging 2021–2030” aims to coordinate global efforts to improve the quality of life for older people, their families, and communities [57]. The WHO promotes from some years ICOPE (Integrated care for older people approach), a simple screening tool to detail intrinsic capacity in older people: in January 2025, the WHO updated the previous version of the manual giving space to some conditions, such as urinary incontinence, not previously considered [58]. In this sense, it seems that more attention is given to interventions promoting intrinsic capacity: in an important paper recently published Beard et al. demonstrated that in England and in China the level of intrinsic capacity is improving, particularly for cognitive, locomotor and vitality domains, probably for broad societal influences and improvements in medical care [59]. Moreover, cohort experiences show that the transition from young to old does not occur uniformly within populations and subgroups: in this regard, superagers are a good example. Superagers have been studied in the context of cognitive resilience, brain structure, and lifestyle factors [60]. Research suggests that these individuals exhibit greater cortical thickness and more resistance to age-related atrophy, particularly in regions associated with memory and executive function (e.g., the anterior cingulate cortex) [61]. Additionally, lifestyle factors such as physical activity, social engagement, and lifelong learning appear to contribute to their exceptional aging trajectories [62].

To promote intrinsic capacity and healthy aging requires a multisectoral approach that considers the healthcare, social, and economic needs of older populations [63]. The key elements of this approach include promoting healthy lifestyle choices, improving access to healthcare services, and adopting policies that promote the social and economic integration of older adults. Public health interventions that promote regular physical activity, balanced nutrition, mental health support, and social connectivity are essential to prevent the onset of chronic diseases and delay functional decline in older people [64, 65]. Regular physical activity, for example, reduces the risk of NCDs, improves mental health, and contributes to a better overall quality of life [66]. Similarly, access to preventive healthcare services, early diagnoses, and the effective management of chronic conditions is crucial to maintaining the health of older populations.

Promoting healthy aging requires a systemic approach involving governments, healthcare providers, communities, and individuals. This approach should aim not only to extend life expectancy but also to improve the quality of life for older adults, ensuring that they can maintain their independence and physical, mental, and social well-being. A central element of this process is the creation of supportive environments that allow older people to remain socially and economically active [67]. Inclusive and well-designed environments can foster independence and autonomy, enabling them to remain socially and economically active, while simultaneously reducing the need for long-term care and improving overall well-being. Ensuring that older adults can live independently is essential to addressing the challenges of population aging and turning them into opportunities for growth and development. Such environments must ensure access to safe infrastructure and adequate healthcare services, as well as promote social support networks that prevent isolation and encourage participation in community life. Several successful initiatives worldwide demonstrate the effectiveness of strategies for healthy aging. For example, Japan’s “Comprehensive Community Care System” integrates healthcare, social, and housing services to reduce hospital admissions among older adults [68]. In Denmark, co-housing communities for older adults combat social isolation, promoting mental well-being and community engagement [69].

At the same time, public health interventions must focus on the prevention of chronic diseases and the delay of functional decline—two key elements for promoting healthy aging. These factors cover a wide range of physical, lifestyle-related, social and psychological aspects [70, 71]. The promotion of healthy lifestyles, such as proper nutrition and regular, adapted physical activity, plays a fundamental role in preventing age-related diseases, improving overall well-being, and maintaining functional independence in later years. Additionally, assistive technologies, personalized geriatric interventions, and home-based care models are instrumental in supporting older adults with chronic conditions, enabling them to maintain autonomy despite health limitations.

Proper nutrition is essential for maintaining the physical and mental health of older adults [72]. As people age, physiological changes, such as a reduction in basal metabolism and the loss of muscle mass, make it crucial to adequately manage caloric and nutritional intake. In particular, obesity among the elderly presents a significant health concern, contributing to the risk of various chronic diseases and functional impairments. Excess body weight can exacerbate age-related decline in mobility and increase the likelihood of conditions such as type 2 diabetes, cardiovascular disease, and certain cancers, adding to the burden on healthcare systems. Adopting dietary models like the Mediterranean diet, which is widely recognized for its long-term benefits, can play a key role in promoting health during aging, by managing weight and improving health outcomes in older populations. Rich in fruits, vegetables, whole grains, fish, legumes, and olive oil, this diet provides essential nutrients and antioxidants that help mitigate obesity-related risks and prevent chronic diseases such as cardiovascular diseases [73], type 2 diabetes [74], certain cancer [75], and neurodegenerative conditions such as dementia and Alzheimer’s [76]. Additionally, the Mediterranean diet has been associated with a reduced risk of depression [77], which is important for the mental well-being of older adults. By focusing on nutrient-dense foods and balanced caloric intake, this dietary approach can help support healthy aging while addressing the growing challenge of obesity in the elderly.

Moreover, proper nutrition in older adults must consider the need to preserve muscle and bone mass. Adequate protein intake is essential to counteract sarcopenia, a common condition involving the loss of muscle mass and strength with aging. The intake of calcium and vitamin D is equally important for bone health, helping to prevent osteoporosis and fractures [78]. Promoting healthy eating habits that include foods rich in high-quality proteins and essential nutrients is therefore a cornerstone for maintaining health and functional independence in older adults [79]. In particular, the new LARN (Reference Levels for Nutrient and Energy Intake), developed by the Italian Society of Human Nutrition (SINU), and literature reviews [80], recognize that the protein requirements for older people are higher compared to younger adults. Specifically, it is suggested to consume at least 1.1–1.2 g of protein per kg of body weight per day, compared to the 0.9 g/kg recommended for younger adults. This increased protein requirement for the older subjects is particularly crucial to prevent sarcopenia and functional decline, which are associated with poorer outcomes of the aging process, reduced physical activity, and chronic diseases. Finally, the new LARN also suggest a higher protein intake when the diet is mainly based on plant-based sources [81].

Regular physical activity is another determining factor for the physical and mental well-being of the older population. Exercise, adapted to the individual’s abilities and health conditions, not only helps maintain musculoskeletal function but also has positive effects on cardiovascular health, metabolism, and mental health [82]. Increasingly, research has shown that regular physical activity also plays a significant role in reducing the risk of cognitive decline and dementia among older adults, as it supports brain health and enhances cognitive resilience. By promoting blood flow to the brain, reducing inflammation, and improving mood, physical activity can help delay or prevent the onset of neurodegenerative conditions, including Alzheimer’s disease. Moreover, studies show that physical activity reduces the risk of developing chronic diseases, such as heart disease, diabetes, and hypertension, as well as improving mood, reducing stress, and preventing depression [83]. A physical activity program for older adults should include resistance exercises to strengthen muscles, aerobic exercises to improve cardiopulmonary capacity, and balance activities to prevent falls, one of the leading causes of morbidity in older adults [84, 85]. It is important that these programs be adapted to individual abilities and follow a gradual approach, respecting the physical limits of older adults while encouraging their progressive improvement. Regular physical activity, when combined with a healthy diet, is one of the most effective strategies to prevent functional decline and promote independence in later years. Even moderate physical activity, such as walking, can significantly reduce the risk of dementia and chronic diseases, ultimately improving quality of life and supporting cognitive health, allowing older adults to remain active and socially engaged [86].

Another fundamental element of public health approaches to healthy aging is timely access to preventive healthcare services. Regular screenings for common diseases in older age, such as diabetes, hypertension, and cardiovascular diseases, are essential for the early diagnosis of conditions that, if left untreated, could lead to severe complications [87]. The effective management of chronic conditions through continuous monitoring and adherence to appropriate therapies helps reduce the burden on healthcare systems and ensures that older adults live longer and with better quality of life [88].

In conclusion, promoting a healthy diet and encouraging regular physical activity are not only tools for preventing age-related diseases but also key elements for maintaining the physical and mental health of older adults, improving their quality of life, and reducing dependence on healthcare services. An integrated and coordinated public health approach is essential to effectively address the challenges posed by population aging and ensure a healthier and more sustainable future for all.

## Public health implications and policy recommendations

The global aging of the population presents complex challenges for healthcare, social, and economic systems worldwide. The public health implications are significant, as they require a restructuring of policies and resources to address the increasing prevalence of chronic diseases and multimorbidity among older adults [89]. Public health interventions must focus on preventing chronic diseases, maintaining functional capacities, and promoting healthy lifestyles, with a strong focus on diet and physical activity, to ensure healthy aging.

Policy recommendations for healthy aging include expanding public health insurance coverage for preventive services, which can reduce the economic burden on older populations by focusing on early intervention [90]. Additionally, the increase in multimorbidity not only imposes a greater burden on healthcare systems but also creates a growing demand for specialized healthcare personnel [91]. Consequently, it is essential to enhance the training and recruitment of healthcare professionals, with particular attention to multidisciplinary teams capable of managing older patients with multiple chronic conditions [91].

As for instance, primary prevention of dementia, which includes strategies aimed at reducing the incidence of dementia before it occurs, is increasingly recognized as an area needing urgent policy focus. While many studies highlight the potential of lifestyle modifications, cognitive engagement, and cardiovascular health management in delaying or preventing dementia onset [92, 93], relatively few policies currently prioritize primary dementia prevention. Incorporating dementia prevention into broader healthy aging policies could significantly improve outcomes for older adults.

To achieve this, policy recommendations for dementia prevention should support accessible screening for cognitive risk factors, encourage physical and mental activity programs tailored for older adults, and promote public awareness campaigns focused on modifiable lifestyle factors, such as diet, exercise, and social engagement, which are associated with cognitive resilience [94]. Expanding research funding for primary prevention strategies and encouraging longitudinal studies will also provide a more robust evidence base for future policies. Additionally, considering the increase in multimorbidity among older adults, it is essential to enhance the training and recruitment of healthcare professionals. Specialized, multidisciplinary teams, particularly those trained in gerontology and dementia care, are crucial to effectively managing the complex needs of patients with both dementia and multiple chronic conditions [95].

Policies should also promote the integration of older adults into social and economic life, including through the adoption of measures that facilitate their continued participation in the workforce in a flexible and dignified manner, while ensuring respect for individual health conditions [57]. Finally, cities and communities must be designed to support healthy aging, ensuring access to healthcare services, safe public spaces, and opportunities for socialization. In this way, the quality of life of older adults can be improved, and the burden on healthcare and social systems can be reduced [96].

In this context, a multisectoral and collaborative approach that involves all levels of society is essential. By adopting this approach, societies can not only address the challenges posed by population aging but also transform them into opportunities for growth and development. Healthy aging can contribute to reducing healthcare costs, increasing economic participation, and improving social cohesion, creating a sustainable and inclusive future for all.

## Conclusion

As populations worldwide continue to age, it is essential to adopt a global approach to healthy aging that addresses the biological, environmental, and social determinants of health. By promoting functional capacity, optimizing intrinsic capacity, and creating supportive environments, societies can ensure that older people live longer, healthier, and more fulfilling lives. In conclusion, the challenges posed by population aging require complex and coordinated public policy responses and interventions. Promoting healthy aging, investing in the prevention of chronic diseases, and strengthening healthcare infrastructure are fundamental strategies to ensure that older adults can enjoy a long, healthy life with a high quality of life, while reducing the pressure on socio-health systems.

## Data Availability

No datasets were generated or analysed during the current study.
